# Deliberation concerning the role of M1-type macrophage subset in oral carcinogenesis

**DOI:** 10.1186/s13046-024-03128-2

**Published:** 2024-08-08

**Authors:** Chen-xi Li, Zhong-cheng Gong, Jing-wen Yu

**Affiliations:** 1 Department of Oral and Maxillofacial Oncology & Surgery, School / Hospital of Stomatology, the First Affiliated Hospital of Xinjiang Medical University, Stomatological Research Institute of Xinjiang Uygur Autonomous Region, Urumqi, 830054 China; 2 Hubei Province Key Laboratory of Oral and Maxillofacial Development and Regeneration, School of Stomatology, Tongji Medical College, Union Hospital, Huazhong University of Science and Technology, Wuhan, 430022 China; 3 Department of Pathology, the First Affiliated Hospital of Xinjiang Medical University, Urumqi, 830054 China

**Keywords:** M1–M2 macrophage polarization, Oral squamous cell carcinoma, Tumor-associated macrophages, *Porphyromonas gingivalis*

## Abstract

Over the last decade, accumulating evidence has suggested that tumor-associated macrophages (TAMs) play a significant role in the tumor development. This commentary wishes to highlight the findings by You, et al. that M1-like TAMs could cascade a mesenchymal/stem-like phenotype of oral squamous cell carcinoma (OSCC) via the IL6/Stat3/THBS1 feedback loop. These unprecedented findings identified M1-like TAMs-regulated processes as potentially tumor-promotion in the context of OSCC immunomicroenvironment.

**Supplementary Information:**

The online version contains supplementary material available at 10.1186/s13046-024-03128-2.

## Background

**Fig. 1:**
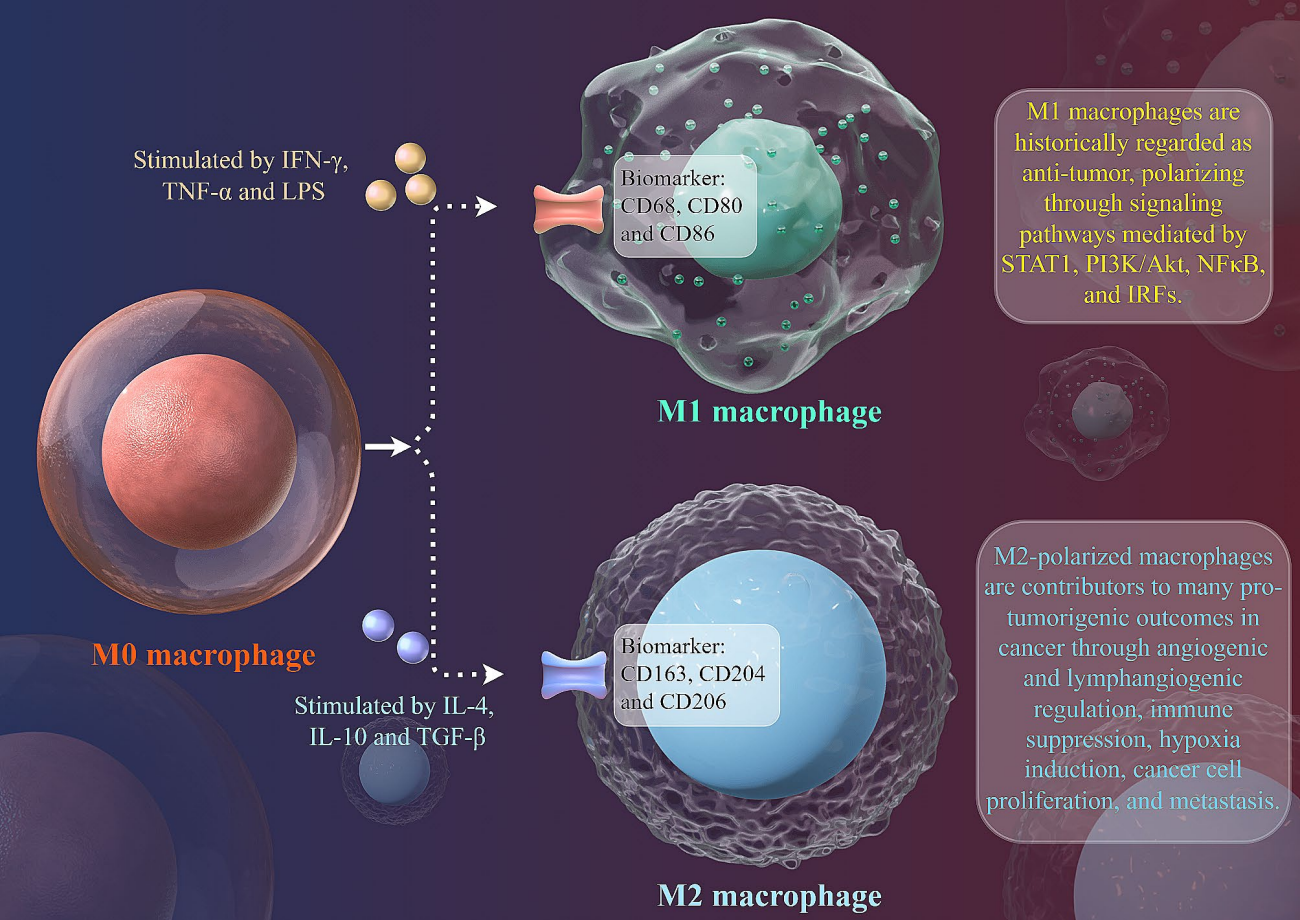
The polarization of TAMs and their characteristics. The illustration displays a general principle of polarized M1-like and M2-like phenotypes. M1-like and M2-like phenotypes represent two extremes of TAMs polarization and display entirely distinct functions. In response to different stimuli in the TME, TAMs undergo M1-like, or M2-like activation. M1-like TAMs are stimulated by IFN-γ, TNF-α, or LPS, express CD68, CD80, and CD86, secrete IL-1β, IL-6, IL-12, IL-23, CXCL9, and CXCL10, and exert anti-tumor effects. In contrast, M2-like TAMs are activated by IL-4, IL-10, or TGF-β, express CD163, CD204, and CD206, secrete IL-10, TNF-α, CCL17, CCL18, CCL22, and CCL24 and promote tumor progression. CCL: chemokine ligand; CXCL: C-X-C motif ligand; IFN: interferon; IL: interleukin; IRF: interferon regulatory factor; LPS: lipopolysaccharide; NF: nuclear factor; PI3K/Akt: phosphatidylinositol 3-kinase/protein kinase B; STAT: signal transducer and activator of transcription; TAMs: tumor-associated macrophages; TGF: transforming growth factor; TME: tumor microenvironment; TNF: tumor necrosis factor. This figure was generated via Figdraw 2.0 online tool (https://www.figdraw.com/#/)

Macrophages are essential elements of both the innate and adaptive immune systems and act as scavengers, modulating the immune response against pathogens and maintaining organism homeostasis. The phagocytic activity of macrophages clears dying and dead cells, and in turn, the clearing of cellular debris provides macrophages with nutrients [1]. Tumor-associated macrophages (TAMs) are crucial components in the tumor microenvironment (TME) and play a fundamental role in tumor development and therapeutic resistance by creating an immunosuppressive microenvironment. Under various cytokine stimulations of circumstances, primary macrophages can be fully polarized into classically activated macrophages (M1) and alternatively activated macrophages (M2), which are the extremes of a continuum of functional states [2, 3] (Fig. 1). Macrophages that infiltrate tumor tissues are frequently driven by tumor-derived cytokines to acquire a polarized M2 phenotype. These functionally polarized cells play a key role in the subversion of adaptive immunity and in inflammatory circuits that promote tumor development and progression [3, 4]. M1 TAMs are thought to be tumoricidal, but a few studies report its pro-tumor role [5].

Intriguingly, a recently published article by You, et al. [6] revealed that the functional roles and associated molecular mechanisms of M1-like TAMs in regulating the malignant progression of oral squamous cell carcinoma (OSCC). According to their investigation, they sought to harvest conditioned media (CM) and exosome supernatants from OSCC cells to activate THP-1 cells and peripheral blood mononuclear cells (PBMCs) derived macrophages exhibiting an M1-like but not M2-like phenotype, in line with the protocol in their previous work [7]. Further analyses indicated that these M1-like TAMs were polarized by taking up OSCC exosome-released THBS1 through p38, Akt, and SAPK/JNK signaling at the early phase. The implications of these M1-like TAMs hinted towards facilitating multiple malignant behaviors of OSCC in vitro assays and in vivo xenograft experiments. More importantly, the results exemplified the complex role of tumor-infiltrating macrophages, because macrophage M1 polarizing effect was turned out to be cancer-promoting rather than conventionally cancer-inhibiting.

**Fig. 2:**
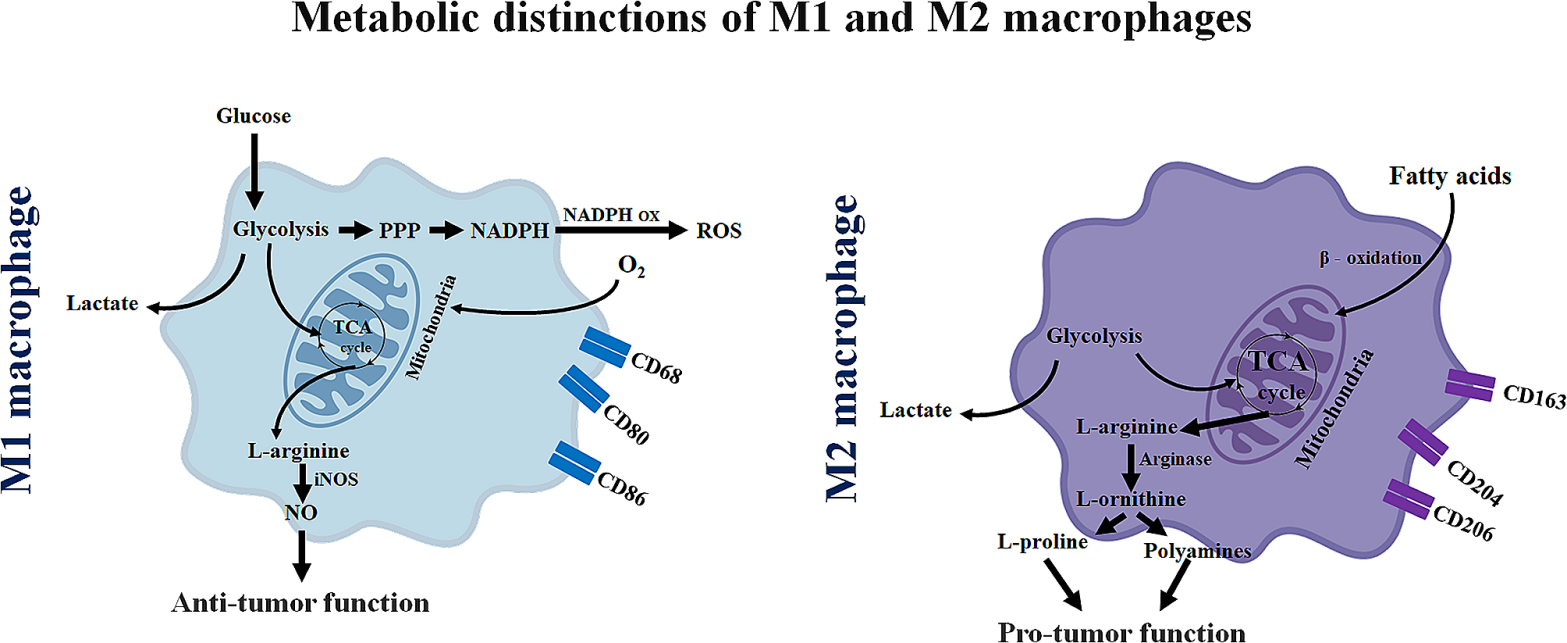
Macrophages undergo a switch in their metabolic pathways that leads to differentiation into either inflammatory (M1) or regulatory (M2) subtypes in response to various cytokine stimulations. While TAMs do not completely follow the M1 and M2 subtypes, they are in general M2-like and facilitate tumor growth by inducing immune suppression. M1-polarized macrophages primarily depend on glucose and the flux of glucose into lactate, ROS production, and NO generation for tumor killing. M2-polarized macrophages primarily depend on beta-oxidation of fatty acids and TCA cycle, while inducing production of polyamines and L-proline to support tumor growth. iNOS: inducible nitric oxide synthase; NADPH: nicotinamide adenine dinucleotide phosphate-reduced form; NADPH ox: NADPH oxidase; NO: nitric oxide; O_2_: oxygen; PPP: pentose phosphate pathway; ROS: reactive oxygen species; TAMs: tumor-associated macrophages; TCA cycle: tricarboxylic acid cycle

Increasing evidence demonstrates that the activation states and diverse spectrum of macrophage subtypes display dynamic heterogeneity in the TME, which plays a critical role in a variety of cancer types, definitely including OSCC. Cancer-derived nanovesicles usually regulate TAMs to differentiate into an anti-inflammatory pro-tumorigenic M2 type not a pro-inflammatory anti-tumorigenic M1 type in the TME [2, 8]. In addition, an established consensus has stated the M2-like TAMs are independent prognostic factors in OSCC based on numerous clinical and preclinical studies [9, 10]. Given the “irregularity” reported the publication by You Y. and colleagues, we cannot help thinking what renders those M1-type TAMs with pro-tumoral effectiveness. Besides, are those M1-type TAMs stably and constantly functioning as accelerator, or have they been induced M1-to-M2 transformation due to metabolic reprogramming in macrophages? Generally, macrophage function and polarization are closely related to altered metabolism. M1 macrophages rely primarily on aerobic glycolysis, whereas M2 macrophages depend on oxidative metabolism (Fig. 2). Nuclear factor (NF)-κB, cyclooxygenase 2, proto-oncogene MYC, Toll-like receptor signaling pathway, Notch signaling pathway and anoxia status are all closely involved in the transition of TAMs from M1 to M2 phenotype [11–13].

**Fig. 3:**
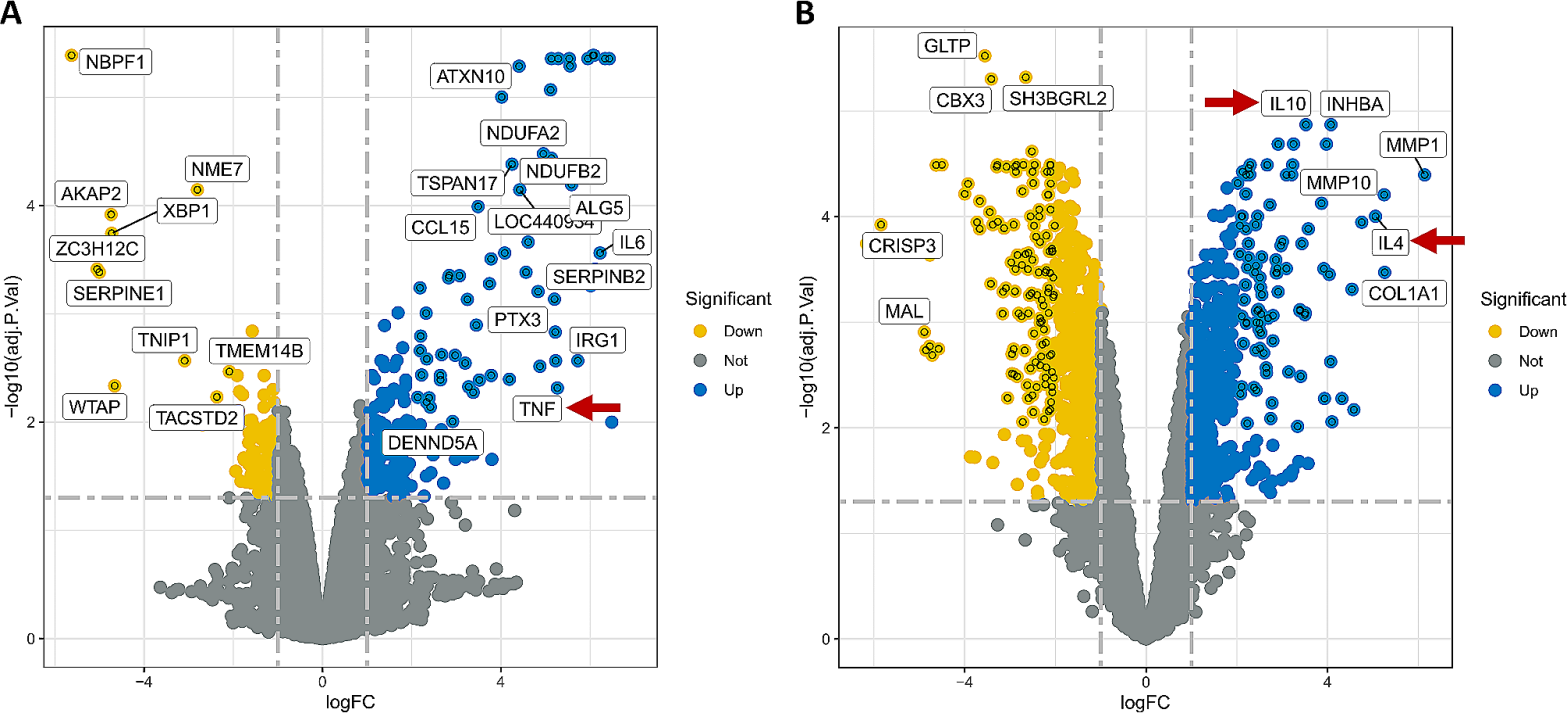
Relationship between OSCC cells and polarized status of TAMs during 36 h co-culture indicated by mRNA sequencing. Transition of TAMs from M1-like to M2-like macrophages could be detected over a period of time. **(A)** Volcanic plot for the mRNA sequencing of M1-like TAMs co-cultured with TSCCA cells at 6 h. **(B)** Volcanic plot for the mRNA sequencing of M2-like TAMs co-cultured with TSCCA cells at 36 h. OSCC: oral squamous cell carcinoma; TAMs: tumor-associated macrophages

According to preliminary results of our ongoing experimental study, hence, we hypothesize some stimuli may contribute to induce the transformation of M1 into M2 TAMs that supports cancer cell growth and metastasis and mediate immunosuppressive effects on the adaptive immune cells of the OSCC TME (Fig. 3, Additional File 1). Moreover, the ratio of M1/M2 macrophages is significantly higher in periodontally affected sites, signifying an imbalance between inflammatory and repair mechanisms; whereas the elevated M2/M1 ratio could successfully predict poor prognosis in OSCC [14]. Furthermore, as a known periodontal pathogen and its presence in orodigestive malignancies [15, 16], *Porphyromonas gingivalis* (*P. gingivalis*) may be considered as one of factors impacting M1-to-M2 transformation. Part of our previous findings showed that a possible mechanism of *P. gingivalis* promoting the progression of OSCC could be regulated by M2-type TAMs polarization via enhancing the expression level of DOK3 (Additional File 2) [17].

## Conclusion

Taken together, although M2 macrophages are generally recognized to have a pro-tumor role, while the effect of M1 macrophages in cancer is controversial. Remarkably, the paper currently being reviewed and discussed is one of the few articles regarding M1-like phenotypic tumor-activator, which reported M1-like TAMs activated by exosome-transferred THBS1 promote malignant behaviors cascading a mesenchymal/stem-like phenotype of OSCC. These data demonstrated that exosomal transfer of THBS1 from oral cancer could polarize macrophages into M1-like TAMs and targeted management of M1-like TAMs shows great potentials for the control of OSCCs, however, there are no ongoing clinical trials. Further clinical outcomes are expected. Besides, further validation is still required to determine the underlying mechanisms for the co-existence of M1 and M2-like TAMs in the TME of OSCC.

### Electronic supplementary material

Below is the link to the electronic supplementary material.

Supplementary Material 1

Supplementary Material 2

Supplementary Material 3
