# Correction: Deliberation concerning the role of M1-type macrophage subset in oral carcinogenesis

**DOI:** 10.1186/s13046-024-03162-0

**Published:** 2024-08-17

**Authors:** Chen-xi Li, Zhong-cheng Gong, Jing-wen Yu

**Affiliations:** 1 Department of Oral and Maxillofacial Oncology & Surgery, School / Hospital of Stomatology, the First Affiliated Hospital of Xinjiang Medical University, Stomatological Research Institute of Xinjiang Uygur Autonomous Region, Urumqi, 830054 China; 2 Hubei Province Key Laboratory of Oral and Maxillofacial Development and Regeneration, School of Stomatology, Tongji Medical College, Union Hospital, Huazhong University of Science and Technology, Wuhan, 430022 China; 3 Department of Pathology, the First Affiliated Hospital of Xinjiang Medical University, Urumqi, 830054 China

**Correction: J Exp Clin Cancer Res 43, 220 (2024)**

10.1186/s13046-024-03128-2

Following publication of the original article [1], the authors found out that Zhong-cheng Gong was not captured as co-corresponding author. The production team failed to capture this during the production process.

The correction does not affect the overall result or conclusion of the article. The original article [1] has been corrected.
