# Supplementary material for: Deliberation concerning the role of M1-type macrophage subset in oral carcinogenesis
Source: J Exp Clin Cancer Res. 2024 Aug 8;43:220. doi: 10.1186/s13046-024-03128-2 (PMC11308489; doi:10.1186/s13046-024-03128-2)
Supplement: Supplementary file 2 — Supplementary Material 2 [file 13046_2024_3128_MOESM2_ESM.docx]

Additional File 1. M1/M2 macrophage ratio in OSCC tissues (n = 200) associated with patients’ clinicopathologic characteristics.

| Variable^†^ | M1/M2 macrophage ratio | | *p*-value |
| --- | --- | --- | --- |
|  | ＜50% (n = 61) | ≥ 50% (n = 139) |  |
| Gender | | | 0.282 |
| Male | 27 (44.3) | 73 (52.5) |  |
| Female | 34 (55.7) | 66 (47.5) |  |
| Age/year | | | 0.580 |
| < 60 | 24 (39.3) | 49 (35.3) |  |
| ≥ 60 | 37 (60.7) | 90 (64.7) |  |
| Periodontal condition | | | 0.000 |
| Well | 28 (45.9) | 28 (20.1) |  |
| Poor | 33 (54.1) | 111 (79.9) |  |
| Survival status | | | 0.002 |
| Alive | 50 (82.0) | 83 (59.7) |  |
| Dead | 11 (18.0) | 56 (40.3) |  |
| T stage | | | 0.000 |
| T1~T2 | 47 (77.0) | 72 (51.8) |  |
| T3~T4 | 14 (23.0) | 67 (48.2) |  |
| N stage | | | 0.025 |
| N0 | 48 (78.7) | 87 (62.6) |  |
| N (+) | 13 (21.3) | 52 (37.4) |  |
| M stage | | | 0.019 |
| M0 | 45 (73.8) | 105 (75.5) |  |
| M1 | 16 (26.2) | 34 (24.5) |  |
| Clinical stage | | | 0.000 |
| I~II | 44 (72.1) | 56 (40.3) |  |
| III~IV | 17 (27.9) | 83 (59.7) |  |
| Recurrence | | | 0.008 |
| No | 54 (88.5) | 115 (82.7) |  |
| Yes | 7 (11.5) | 24 (17.3) |  |
| Tumor size/cm | | | 0.014 |
| < 3 | 36 (59.0) | 55 (39.6) |  |
| ≥ 3 | 25 (41.0) | 84 (60.4) |  |
| Differentiation | | | 0.003 |
| Well | 49 (80.33) | 80 (57.6) |  |
| Moderate | 11 (18.03) | 38 (27.3) |  |
| Poor | 1 (1.64) | 21 (15.1) |  |
| M2-macrophage infiltration | | | 0.008 |
| Yes | 24 (39.3) | 83 (59.7) |  |
| No | 37 (60.7) | 56 (40.3) |  |

^†^ Clinicopathologic characteristics of included patients were described as absolute frequency (percentage), and bivariate analysis to evaluate the association between clinicopathologic variables and the ratio of M1/M2 macrophages in OSCC immunomicroenvironment was determined using the Chi-square or Fisher's exact test.

OSCC: oral squamous cell carcinoma


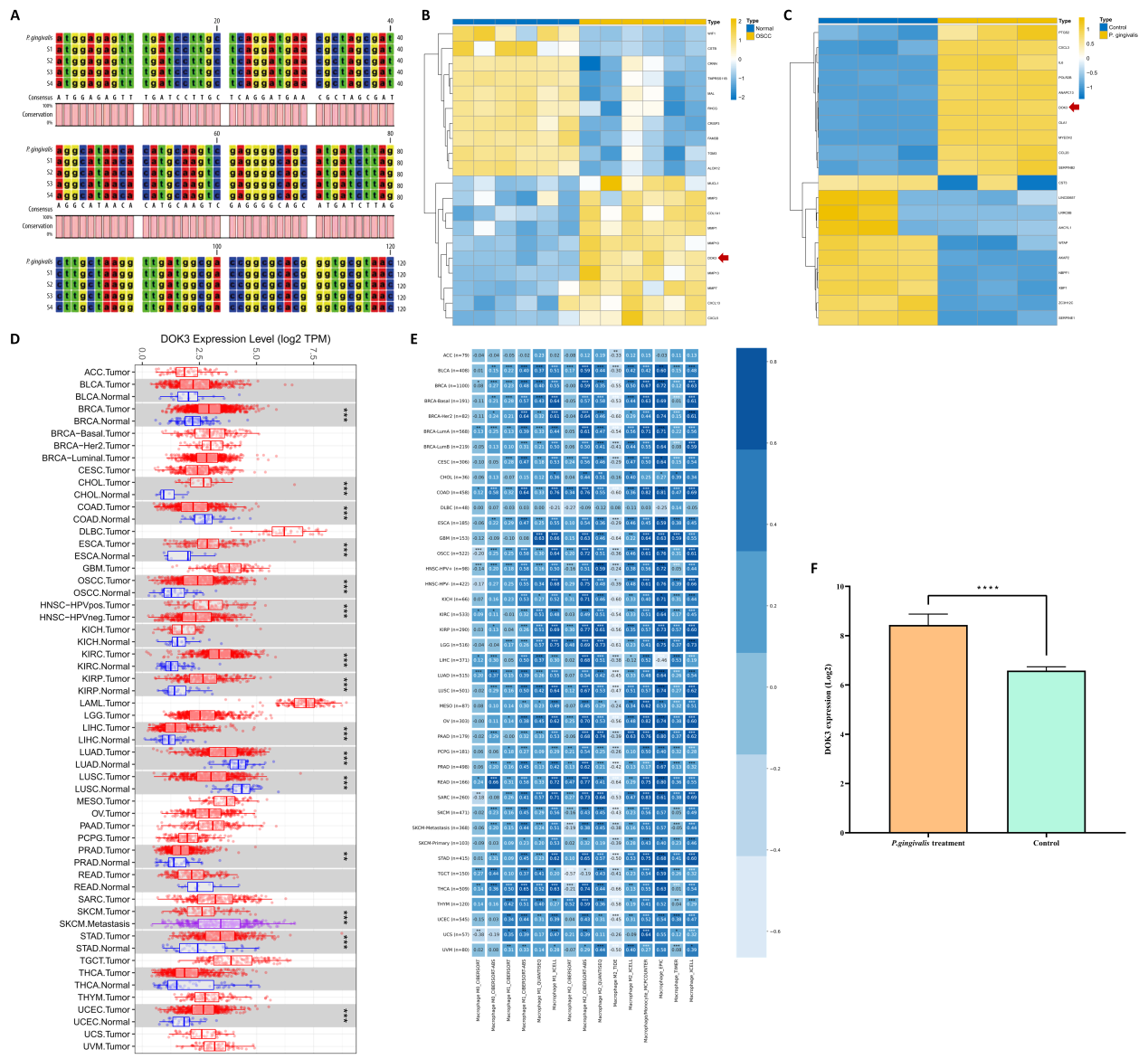


Additional File 2. **A.** 16S rDNA sequencing alignment of *P. gingivalis* detected and determined in OSCC samples. **B.** cDEGs between OSCC (6 cases) and matched normal tissue (6 cases); **C.** DEGs between *P. gingivalis*-positive (3 cases) and *gingivalis*-negative (3 cases) in 6 OSCC samples. **D.** The differential expression map of DOK3 on pan-cancer data showing a significant increase in OSCC than that in normal tissue. **E.** Correlation between DOK3 and infiltrating tumor-associated macrophage. **F.** DOK3 expression in *P.* *gingivalis* infection of macrophages microarray. Statistical differences were considered significant if * *p* < 0.05; ** *p* < 0.01; *** *p* < 0.001; **** *p* < 0.0001. cDEGs: co-differentially-expressed genes; DOK3: downstream of tyrosine kinase 3/docking protein 3; OSCC: oral squamous cell carcinoma; *P. gingivalis*: *Porphyromonas gingivalis*.

Separate Figure A was published in our previous article (please see <https://doi.org/10.12659/MSM.937126>.), and its publication copyright and the re-use license has been obtained.
