# Supplementary material for: Deliberation concerning the role of M1-type macrophage subset in oral carcinogenesis
Source: J Exp Clin Cancer Res. 2024 Aug 8;43:220. doi: 10.1186/s13046-024-03128-2 (PMC11308489; doi:10.1186/s13046-024-03128-2)
Supplement: Supplementary file 3 — Supplementary Material 3 [file 13046_2024_3128_MOESM3_ESM.docx]

**Deliberation concerning the role of M1-type macrophage subset in oral carcinogenesis**

**Comment on “M1-like tumor-associated macrophages cascade a mesenchymal/stem-like phenotype of oral squamous cell carcinoma via the IL6/Stat3/THBS1 feedback loop”**

Chen-xi Li (DDS, MD, PhD)^a,b^, Zhong-cheng Gong (DDS, MD, PhD)^a^, Jing-wen Yu (MD)^c^

^a^ Department of Oral and Maxillofacial Oncology & Surgery, the First Affiliated Hospital of Xinjiang Medical University, School / Hospital of Stomatology, Stomatological Research Institute of Xinjiang Uygur Autonomous Region. Urumqi 830054, China

^b^ Hubei Province Key Laboratory of Oral and Maxillofacial Development and Regeneration, School of Stomatology, Tongji Medical College, Union Hospital, Huazhong University of Science and Technology. Wuhan 430022, China

^c^ Department of Pathology, the First Affiliated Hospital of Xinjiang Medical University. Urumqi 830054, China

**Address correspondence to:** Dr. Chen-xi Li, ORCID ID: 0000-0003-1443-4769

Department of Oral and Maxillofacial Oncology & Surgery, School / Hospital of Stomatology, the First Affiliated Hospital of Xinjiang Medical University. No. 137 Liyushan South Road, Urumqi 830054, P.R.China.

Email: [lichenxiuke@gmail.com](mailto:lichenxiuke@gmail.com); Fax: +86 991-4366081

Co-corresponding Author: Prof. Zhong-cheng Gong, ORCID ID: 0000-0002-1871-1500

Department of Oral and Maxillofacial Oncology & Surgery, School / Hospital of Stomatology, the First Affiliated Hospital of Xinjiang Medical University. No. 137 Liyushan South Road, Urumqi 830054, P.R.China.

Email: [gzc740904@xjmu.edu.cn](mailto:gzc740904@xjmu.edu.cn%20); Fax: +86 991-4366079

**Abstract**

Over the last decade, accumulating evidence has suggested that tumor-associated macrophages (TAMs) play a significant role in the tumor development. This commentary wishes to highlight the findings by You, et al. that M1-like TAMs could cascade a mesenchymal/stem-like phenotype of oral squamous cell carcinoma (OSCC) via the IL6/Stat3/THBS1 feedback loop. These unprecedented findings identified M1-like TAMs-regulated processes as potentially tumor-promotion in the context of OSCC immunomicroenvironment.

**Keywords:** M1–M2 macrophage polarization; Oral squamous cell carcinoma; Tumor-associated macrophages; *Porphyromonas gingivalis*

**Background**

Macrophages are essential elements of both the innate and adaptive immune systems and act as scavengers, modulating the immune response against pathogens and maintaining organism homeostasis. The phagocytic activity of macrophages clears dying and dead cells, and in turn, the clearing of cellular debris provides macrophages with nutrients [1]. Tumor-associated macrophages (TAMs) are crucial components in the tumor microenvironment (TME) and play a fundamental role in tumor development and therapeutic resistance by creating an immunosuppressive microenvironment. Under various cytokine stimulations of circumstances, primary macrophages can be fully polarized into classically activated macrophages (M1) and alternatively activated macrophages (M2), which are the extremes of a continuum of functional states [2,3] (Fig. 1).Macrophages that infiltrate tumor tissues are frequently driven by tumor-derived cytokines to acquire a polarized M2 phenotype. These functionally polarized cells play a key role in the subversion of adaptive immunity and in inflammatory circuits that promote tumor development and progression [3,4]. M1 TAMs are thought to be tumoricidal, but a few studies report its pro-tumor role [5].

Intriguingly, a recently published article by You, et al. [6] revealed that the functional roles and associated molecular mechanisms of M1-like TAMs in regulating the malignant progression of oral squamous cell carcinoma (OSCC). According to their investigation, they sought to harvest conditioned media (CM) and exosome supernatants from OSCC cells to activate THP-1 cells and peripheral blood mononuclear cells (PBMCs) derived macrophages exhibiting an M1-like but not M2-like phenotype, in line with the protocol in their previous work [7]. Further analyses indicated that these M1-like TAMs were polarized by taking up OSCC exosome-released THBS1 through p38, Akt, and SAPK/JNK signaling at the early phase. The implications of these M1-like TAMs hinted towards facilitating multiple malignant behaviors of OSCC *in vitro* assays and *in vivo* xenograft experiments. More importantly, the results exemplified the complex role of tumor-infiltrating macrophages, because macrophage M1 polarizing effect was turned out to be cancer-promoting rather than conventionally cancer-inhibiting.

Increasing evidence demonstrates that the activation states and diverse spectrum of macrophage subtypes display dynamic heterogeneity in the TME, which plays a critical role in a variety of cancer types, definitely including OSCC. Cancer-derived nanovesicles usually regulate TAMs to differentiate into an anti-inflammatory pro-tumorigenic M2 type not a pro-inflammatory anti-tumorigenic M1 type in the TME [2,8]. In addition, an established consensus has stated the M2-like TAMs are independent prognostic factors in OSCC based on numerous clinical and preclinical studies [9,10]. Given the “irregularity” reported the publication by You Y. and colleagues, we cannot help thinking what renders those M1-type TAMs with pro-tumoral effectiveness. Besides, are those M1-type TAMs stably and constantly functioning as accelerator, or have they been induced M1-to-M2 transformation due to metabolic reprogramming in macrophages? Generally, macrophage function and polarization are closely related to altered metabolism. M1 macrophages rely primarily on aerobic glycolysis, whereas M2 macrophages depend on oxidative metabolism (Fig. 2). Nuclear factor (NF)-κB, cyclooxygenase 2, proto-oncogene MYC, Toll-like receptor signaling pathway, Notch signaling pathway and anoxia status are all closely involved in the transition of TAMs from M1 to M2 phenotype [11-13].

According to preliminary results of our ongoing experimental study, hence, we hypothesize some stimuli may contribute to induce the transformation of M1 into M2 TAMs that supports cancer cell growth and metastasis and mediate immunosuppressive effects on the adaptive immune cells of the OSCC TME (Fig. 3, Additional File 1). Moreover, the ratio of M1/M2 macrophages is significantly higher in periodontally affected sites, signifying an imbalance between inflammatory and repair mechanisms; whereas the elevated M2/M1 ratio could successfully predict poor prognosis in OSCC [14]. Furthermore, as a known periodontal pathogen and its presence in orodigestive malignancies [15,16], *Porphyromonas gingivalis* (*P. gingivalis*) may be considered as one of factors impacting M1-to-M2 transformation. Part of our previous findings showed that a possible mechanism of *P. gingivalis* promoting the progression of OSCC could be regulated by M2-type TAMs polarization via enhancing the expression level of DOK3 (Additional File 2) [17].

**Conclusion**

Taken together, although M2 macrophages are generally recognized to have a pro-tumor role, while the effect of M1 macrophages in cancer is controversial. Remarkably, the paper currently being reviewed and discussed is one of the few articles regarding M1-like phenotypic tumor-activator, which reported M1-like TAMs activated by exosome-transferred THBS1 promote malignant behaviors cascading a mesenchymal/stem-like phenotype of OSCC. These data demonstrated that exosomal transfer of THBS1 from oral cancer could polarize macrophages into M1-like TAMs and targeted management of M1-like TAMs shows great potentials for the control of OSCCs, however, there are no ongoing clinical trials. Further clinical outcomes are expected. Besides, further validation is still required to determine the underlying mechanisms for the co-existence of M1 and M2-like TAMs in the TME of OSCC.

***Declarations***

**Conflict of interest** No potential conflict of interest was reported by the author(s).

**Funding**

This work was funded by National Natural Science Foundation of China (No. 82360481); Open Project of Hubei Province Key Laboratory of Oral and Maxillofacial Development and Regeneration (No. 2022kqhm008); Xinjiang Postgraduate Scientific Research Innovation Project (No. XJ2023G174).

**Author Contributions Statement**

C. Li wrote the original draft and revised the manuscript. Z. Gong and J. Yu reviewed the manuscript.

**Acknowledgements**

Authors appreciate all financial support that granted, and are also grateful to the research volunteers and collaborators. Many thanks for Sonnig Biomedical Studio, Urumqi, China as that team was so kind to help run the sequencing procedure. This article is invited to be presented in the coming The 4th National Academic Annual Conference on Oral and Maxillofacial Head and Neck Oncology held by the Professional Committee of Oral and Maxillofacial Head and Neck Oncology of the Chinese Stomatological Association (Hohhot, Jul. 5th-7th, 2024).

**References**

1. Lazarov T, Juarez-Carreño S, Cox N, Geissmann F. Physiology and diseases of tissue-resident macrophages. Nature. 2023;618(7966):698-707.

2. Li CX, Gong ZC, Zheng NB. Progress in the regulatory effects of tumor-derived exosomes on macrophage polarization. Chin J Microbiol Immunol. 2023;43(01):76-83.

3. Christofides A, Strauss L, Yeo A, Cao C, Charest A, Boussiotis VA. The complex role of tumor-infiltrating macrophages. Nat Immunol. 2022;23(8):1148-1156.

4. Li M, Yang Y, Xiong L, Jiang P, Wang J, Li C. Metabolism, metabolites, and macrophages in cancer. J Hematol Oncol. 2023;16(1):80.

5. Zong Z, Zou J, Mao R, Ma C, Li N, Wang J, et al. M1 Macrophages Induce PD-L1 Expression in Hepatocellular Carcinoma Cells Through IL-1β Signaling. Front Immunol. 2019;10:1643.

6. You Y, Tian Z, Du Z, Wu K, Xu G, Dai M, et al. M1-like tumor-associated macrophages cascade a mesenchymal/stem-like phenotype of oral squamous cell carcinoma via the IL6/Stat3/THBS1 feedback loop. J Exp Clin Cancer Res. 2022;41(1):10.

7. Xiao M, Zhang J, Chen W, Chen W. M1-like tumor-associated macrophages activated by exosome-transferred THBS1 promote malignant migration in oral squamous cell carcinoma. J Exp Clin Cancer Res. 2018;37(1):143.

8. Baig MS, Roy A, Rajpoot S, Liu D, Savai R, Banerjee S, et al. Tumor-derived exosomes in the regulation of macrophage polarization. Inflamm Res. 2020;69(5):435-451.

9. Bruna F, Scodeller P. Pro-Tumorigenic Macrophage Infiltration in Oral Squamous Cell Carcinoma and Possible Macrophage-Aimed Therapeutic Interventions. Front Oncol. 2021;11:675664.

10. Kiseleva V, Vishnyakova P, Elchaninov A, Fatkhudinov T, Sukhikh G. Biochemical and molecular inducers and modulators of M2 macrophage polarization in clinical perspective. Int Immunopharmacol. 2023;122:110583.

11. Wang S, Liu R, Yu Q, Dong L, Bi Y, Liu G. Metabolic reprogramming of macrophages during infections and cancer. Cancer Lett. 2019;452:14-22.

12. Mehla K, Singh PK. Metabolic Regulation of Macrophage Polarization in Cancer. Trends Cancer. 2019;5(12):822-834.

13. Larionova I, Kazakova E, Patysheva M, Kzhyshkowska J. Transcriptional, Epigenetic and Metabolic Programming of Tumor-Associated Macrophages. Cancers (Basel). 2020;12(6):1411.

14. Li CX, Su Y, Gong ZC, Liu H. *Porphyromonas gingivalis* Activation of Tumor-Associated Macrophages via DOK3 Promotes Recurrence of Oral Squamous Cell Carcinoma. Med Sci Monit. 2022;28:e937126.

15. Li CX, Li MQ, Wei W, Gong ZC, Tan XR, Liu H, et al. Evidence-based evaluation regarding the impact of *Porphyromonas gingivalis* on the malignancies of digestive system and its possible pathogenic mechanism. J Dig Oncol. 2023;15(4):346-357.

16. Li CX, Yu JW, Gong ZC. Probability Regarding the Tumor-Promoter Role of *Porphyromonas gingivalis* for the Gastric Carcinogenesis. Dig Dis Sci. 2024;69(6):2260-2261.

17. Li C, Li M, Wei W, Wang Z, Yu J, Gong Z. Association of DOK3 and infiltrated tumor-associated macrophages with risk for the prognosis of *Porphyromonas gingivalis*-infected oral cancer: a 12-year data analysis of 200 patients from a tertiary teaching hospital, Urumqi, China. BMC Cancer. 2024;24(1):534.

**Figure legends**

Figure 1. The polarization of TAMs and their characteristics. The illustration displays a general principle of polarized M1-like and M2-like phenotypes. M1-like and M2-like phenotypes represent two extremes of TAMs polarization and display entirely distinct functions. In response to different stimuli in the TME, TAMs undergo M1-like, or M2-like activation. M1-like TAMs are stimulated by IFN-γ, TNF-α, or LPS, express CD68, CD80, and CD86, secrete IL-1β, IL-6, IL-12, IL-23, CXCL9, and CXCL10, and exert anti-tumor effects. In contrast, M2-like TAMs are activated by IL-4, IL-10, or TGF-β, express CD163, CD204, and CD206, secrete IL-10, TNF-α, CCL17, CCL18, CCL22, and CCL24 and promote tumor progression. CCL: chemokine ligand; CXCL: C-X-C motif ligand; IFN: interferon; IL: interleukin; IRF: interferon regulatory factor; LPS: lipopolysaccharide; NF: nuclear factor; PI3K/Akt: phosphatidylinositol 3-kinase/protein kinase B; STAT: signal transducer and activator of transcription; TAMs: tumor-associated macrophages; TGF: transforming growth factor; TME: tumor microenvironment; TNF: tumor necrosis factor.

This figure was generated via Figdraw 2.0 online tool (<https://www.figdraw.com/#/>).

Figure 2. Macrophages undergo a switch in their metabolic pathways that leads to differentiation into either inflammatory (M1) or regulatory (M2) subtypes in response to various cytokine stimulations. While TAMs do not completely follow the M1 and M2 subtypes, they are in general M2-like and facilitate tumor growth by inducing immune suppression. M1-polarized macrophages primarily depend on glucose and the flux of glucose into lactate, ROS production, and NO generation for tumor killing. M2-polarized macrophages primarily depend on beta-oxidation of fatty acids and TCA cycle, while inducing production of polyamines and L-proline to support tumor growth. iNOS: inducible nitric oxide synthase; NADPH: nicotinamide adenine dinucleotide phosphate-reduced form; NADPH ox: NADPH oxidase; NO: nitric oxide; O_2_: oxygen; PPP: pentose phosphate pathway; ROS: reactive oxygen species; TAMs: tumor-associated macrophages; TCA cycle: tricarboxylic acid cycle.

Figure 3. Relationship between OSCC cells and polarized status of TAMs during 36h co-culture indicated by mRNA sequencing. Transition of TAMs from M1-like to M2-like macrophages could be detected over a period of time. **A.** Volcanic plot for the mRNA sequencing of M1-like TAMs co-cultured with TSCCA cells at 6h. **B.** Volcanic plot for the mRNA sequencing of M2-like TAMs co-cultured with TSCCA cells at 36h. OSCC: oral squamous cell carcinoma; TAMs: tumor-associated macrophages.
