# Supplementary material for: Deliberation concerning the role of M1-type macrophage subset in oral carcinogenesis
Source: J Exp Clin Cancer Res. 2024 Aug 8;43:220. doi: 10.1186/s13046-024-03128-2 (PMC11308489; doi:10.1186/s13046-024-03128-2)
Supplement: Supplementary file 1 — Supplementary Material 1 [file 13046_2024_3128_MOESM1_ESM.docx]

May 30^th^, 2024

Prof. Mauro Castelli, PhD

Editor-in-Chief

*Journal of Experimental & Clinical Cancer Research*

Dear editors:

I wish to resubmit our manuscript, entitled “Deliberation concerning the role of M1-type macrophage subset in oral carcinogenesis

Comment on “M1-like tumor-associated macrophages cascade a mesenchymal/stem-like phenotype of oral squamous cell carcinoma via the IL6/Stat3/THBS1 feedback loop”” The Manuscript Number is **JECC-D-24-00960**.

We thank you and the reviewers for your thoughtful insights and suggestions. The manuscript has benefited from these insightful suggestions. I look forward to working with you and the reviewers to move this manuscript closer to publication in prestigious *Journal of Experimental & Clinical Cancer Research*.

Reviewer reports:

Reviewer #1: 1. What is the source of 16S rDNA sequencing data?
**Response:** Thank you for your careful reading and kind suggestion. *Porphyromonas gingivalis* DNA was detected using the specimens from 50 patients with OSCC admitted to authors’ affiliation between January 2020 and June 2022, as described in our previous publication (please see https://doi.org/10.12659/MSM.937126).

2. Some letters in Figure 2 are too small to be seen;
3. The author suggests that Pg promotes the M1/M2 ratio, but later they states that Pg affects the M1-M2 conversion. These two points seem to contradict each other;
4. The views on metabolism and Pg infection in the article seem somewhat disconnected, and we are also curious whether Pg could affect TAM polarization towards sub-typing through metabolism.
5. The part of Pg infection in OSCC is limited, and there is a significant mismatch between it and the description of the Title.
**Response to comments 2 to 5:** Thank you for your careful reading and kind suggestion. We have re-designed and re-written the full text. Please review the revised manuscript.


Reviewer #2: Li et al. commented on the promotion of OSCC progression by Pg and the paradoxical promotion of tumor progression by IL-6 secreted by M1-like TAMs, which can arouse wide interest among researchers. The authors hypothesized the relationship between Pg and M1 in promoting tumor progression, leading researchers to further explore the link between the them. However, there are still some details that need to be revised.


Article writing should be strengthened
**Response:** We improve the manuscript with an English native speaker (see *Acknowledgement* section) as well as Springer Nature Author Services. Please find the certificate attached.

The discussion of macrophages seems to be somewhat redundant, while TAMs, especially M1 TAMs, are not sufficiently addressed.
MYC, etc., should not be placed with anoxia status.
TAMs are strongly influenced by tumor cells, but the role of other cells, such as CAFs, on TAMs should not be ignored.
Fig1, M0, M1, M2 and other fonts are unified. For those readers who are challenged with color discrimination, please change the red and green color scheme in the figure.
INF-r, IL-4 and other marks are easy to be misunderstood, please further correct.
As for Fig.2, I have concerns about the copyright. Please indicate in the legend the ownership of the copyright and whether the use license has been obtained. **Response:** Thank you for your careful reading and kind suggestion. We have re-designed and re-written the full text. Please review the revised submission.

We have also revised the format based on a recent *Journal of Experimental & Clinical Cancer Research* issue to conform to Journal style.

Thank you for your time. I look forward to hearing from you.

Sincerely,

Dr. Chen-xi Li

Department of Oral and Maxillofacial Oncology & Surgery, the First Affiliated Hospital of Xinjiang Medical University, School / Hospital of Stomatology, Stomatological Research Institute of Xinjiang Uygur Autonomous Region. No.137 Liyushan South Road, Urumqi 830054, P.R. China.

E-mail: [lichenxiuke@gmail.com](mailto:lichenxiuke@gmail.com)

Fax: +86 991-4366081
